# Two words as one: A multi-naming investigation of the age-of-acquisition effect in compound-word processing

**DOI:** 10.3758/s13421-019-00986-6

**Published:** 2019-11-21

**Authors:** Mahmoud Medhat Elsherif, Jon C. Catling, Steven Frisson

**Affiliations:** School of Psychology, University of Birmingham, 52 Pritchatts Road, Birmingham, B15 2TT UK

**Keywords:** Age-of-acquisition, Word naming, Combinatorial naming, Compound words

## Abstract

Previous research has shown that early-acquired words are produced faster than late-acquired words. Juhasz and colleagues (Juhasz, Lai & Woodcock, *Behavior Research Methods, 47* (4), 1004-1019, 2015; Juhasz, *The Quarterly Journal of Experimental Psychology*, 1-10, 2018) argue that the Age-of-Acquisition (AoA) loci for complex words, specifically compound words, are found at the lexical/semantic level. In the current study, two experiments were conducted to evaluate this claim and investigate the influence of AoA in reading compound words aloud. In Experiment 1, 48 participants completed a word naming task. Using general linear mixed modelling, we found that the age at which the compound word was learned significantly affected the naming latencies beyond the other psycholinguistic properties measured. The second experiment required 48 participants to name the compound word when the two morphemes were presented with a space in-between (combinatorial naming, e.g. *air plane*). We found that the age at which the compound word was learned, as well as the AoA of the individual morphemes that formed the compound word, significantly influenced combinatorial naming latency. These findings are discussed in relation to theories of the AoA in language processing.

**Electronic supplementary material:**

The online version of this article (10.3758/s13421-019-00986-6) contains supplementary material, which is available to authorized users.

## Introduction

The Age-of-Acquisition (AoA) of the stimuli (e.g. Arnon, McCauley & Christiansen, 2017; Carroll & White, 1973) is one of the variables that have been shown to affect retrieval time for picture names, words and multi-word units. The AoA effect refers to the age at, or order in which, one learns a word. The effect is such that words that have been learnt earlier in life are significantly easier to retrieve than words learnt later in life (e.g. Juhasz, 2005). The effect of AoA has been shown in a variety of tasks such as word naming (e.g. Ellis & Morrison, 1998), picture naming (e.g. Barry, Hirsch, Johnston, & Williams, 2001), naming-to-definition (Navarrete, Pastore, Valentini & Peressotti, 2015), phrasal lexical decision (Arnon et al., 2017) and picture-word verification (e.g. Catling & Johnston, 2009). For more details, see reviews by Johnston and Barry (2006) and Juhasz (2005), who discuss the effect of AoA in different methodologies, neurotypical and clinical samples and in different languages. The current study investigates the role of the AoA in the production of compound words. Specifically, we consider the importance of lexical(whole-word)/semantic and morpheme predictors of AoA on word naming and combinatorial naming tasks.

## Theories of the Age-of-Acquisition (AoA) effects

Three theories seek to explain the structure and function of the AoA effects. The semantic hypothesis, originally posited by van Loon-Vervoon (1989, cited in Brysbaert, van Wijnendaele & De Deyne, 2000), argues that conceptual knowledge is dependent on the order in which the concepts are learned. The speed and efficiency with which the concept in the semantic system is accessed is determined by the order of acquisition (Henry & Kuperman, 2013; Steyvers & Tenebaum, 2005). According to this account, early-learned concepts have acquired more semantic connections and are placed at the centre of the network, making them more easily accessible (e.g. Steyvers & Tenebaum, 2005). Evidence for this hypothesis comes from the finding that AoA effects were shown in an animacy decision task (e.g. Raling, Hanne, Schroder, Kebler & Wartenburger, 2017). Raling et al. (2017) asked participants to categorise written words as either living or non-living. The findings revealed the main effects of AoA, typicality and semantic domain, with no interactions between these variables, enabling the authors to conclude that the AoA effects were at a semantic level. Evidence to support the semantic hypothesis has also been provided in studies that use a category verification task (Raling, Holzgrefe-Lang, Schroder & Wartenburger, 2015), text reading (Juhasz & Rayner, 2003, 2006) and a semantic Simon task (Ghyselinck, Custers & Brysbaert, 2004). The semantic Simon task requires participants to decide whether a stimulus word is printed in upper- or lower-case letters. They must verbally make a response (i.e. ‘living’ or ‘non-living’) that is either congruent with the meaning of the word (e.g. saying ‘living’ to the stimulus ‘GORILLA’) or incongruent (e.g. saying ‘non-living’ to the stimulus ‘GORILLA’). Early-acquired words showed a stronger effect of semantic congruency, illustrating that the meaning of early-acquired words was activated faster. Hence, Ghyselinck et al. concluded that semantics plays an important role in the AoA effect.

The semantic locus can be subsumed under a multiple-loci account of the AoA effect. According to this account, the AoA effect is situated at multiple locations within the object and word processing systems (i.e. orthographic, semantic and phonological levels; Moore, Smith-Spark & Valentine, 2004). The magnitude of the effect varies depending on the number of levels of processing required to perform a particular task (Catling & Johnston, 2009). Catling and Johnston (2009) demonstrated that the AoA effect increases as more connections between processing levels are activated during the task. Evidence to support the multiple-loci hypothesis has also been illustrated in studies that use an object classification task (Catling & Johnston, 2006), a name-object verification task (e.g. Preece, 2015), picture naming (Kittredge, Dell, Verkuilen & Schwartz, 2008), degraded picture naming (Catling, Dent & Williamson, 2008), delayed picture naming (Navarrete, Scaltritti, Mulatti & Peressotti, 2013), and an object decision task with articulatory suppression (Holmes & Ellis, 2006). The latter involves participants making a decision as to whether an object is real or not, while repeating the word ‘supercalifragilisticexpialidocious’. If the AoA phenomenon is phonological, the AoA effect should not be shown. Holmes and Ellis found that although the AoA effect was robust, its magnitude was reduced. These findings showed that the AoA effect may have multiple loci, including at the phonological level.

The multiple-loci account also overlaps with the arbitrary mapping (AM) account of AoA (Ellis & Lambon Ralph, 2000; Lambon Ralph & Ehsan, 2006). The AM account posits that the AoA effects reflect the arbitrary nature of mapping between input (e.g. orthography) and output (e.g. phonological or semantic representations). According to the AM hypothesis, prior to the entry of early-acquired items, the neural network is plastic. Early-acquired items benefit from the plasticity of the network, which leads to these items having a rich and stable representation that is better consolidated in the mental lexicon. Next, early-acquired items begin to adjust the connections or weights between the input and output representations, causing the network to lose plasticity and, in turn, making it difficult for late-acquired items to be consolidated. This results in a processing advantage for early-acquired items over late-acquired items. Put simply, early-acquired items have a larger influence on the network’s final structure. Larger effects of AoA are demonstrated when the mapping between input and output is arbitrary (e.g. between orthography/phonology and semantics, as in picture naming) than when the mapping between input and output is more systematic and regular (e.g. between orthography and phonology, as in word naming; see reviews by Juhasz, 2005; Johnston & Barry, 2006). The AoA effect may therefore be focused on the connections between inputs (e.g. orthography, phonology, visual features) and outputs (e.g. semantics) instead of an individual system such as the semantic system. Similar to the multiple-loci account, the AM hypothesis places the effect of AoA at multiple levels: perceptual, semantic, and/or phonological. Evidence supporting this hypothesis includes picture naming and word naming tasks with the same stimuli (Lambon Ralph & Ehsan, 2006), in which the AoA effects were found to be larger in the picture naming than word naming tasks. Additional evidence for the AM hypothesis has been demonstrated with learning tasks that focus on the order of acquisition effect. Over multiple sessions, items that are learned earlier are processed faster than items that are learned later, even when frequency of exposure is controlled (Catling, Dent, Preece & Johnston, 2013; Joseph, Wonnacott, Forbes & Nation, 2014; Stewart & Ellis, 2008). Similar to picture-naming tasks, these learning tasks rely on the arbitrary mapping between input (i.e. orthographic or perceptual depending on type of stimuli) and output (i.e. semantic and phonological knowledge).

## The AoA effect in complex words

Although the effects of AoA have been shown within a plethora of tasks and several languages, investigations into the effects of AoA beyond monomorphemic words are relatively scarce (e.g. phrases: Arnon, et al., 2017; compound words: Juhasz, 2018; Juhasz, Lai & Woodcock, 2015). Relative to polysyllabic words, only a small minority of words are monosyllabic in English, while many other languages hardly possess monosyllabic words (Mousikou, Sadat, Lucas & Rastle, 2017). When examining a sample of 15 studies conducted in English (e.g. Belke, Brysbaert, Meyer & Ghyselinck, 2005; Catling & Johnston, 2009; Navarette et al., 2015), we found that 10-15% of the items used were compound words (e.g. butterfly).

This focus on monosyllabic words, and the possible confound in item selection, might at least partially explain the current disagreement as to whether semantic effects are genuine or not. For monosyllabic words, the evidence for a semantic influence is mixed. In monomorphemic word naming tasks, significant effects of imageability, a semantic variable, have been found when reading aloud low-frequency and irregular words in English (Strain Patterson, & Seidenberg, 1995, 2002; but see replication by Monaghan & Ellis, 2002). In studies of transparent orthographies such as Italian, no effect of imageability is observed (Barca, Burani & Arduino, 2002). Results from a megastudy by Cortese, Yates, Schock and Viliks (2018) showed that imageability affected word recognition in conditional word naming (i.e. name the word, not nonword) and lexical decision tasks but not in word naming. They concluded that semantic information is important for making a lexical decision, but not crucial for orthographic-to-phonological translation. This indicates that semantics may not be key for spelling-sound correspondence.

In contrast to the involvement of semantics in monomorphemic words, the involvement of semantics in multisyllabic and morphologically complex words such as compound words have been repeatedly shown. Yap and Balota (2009) extracted the naming latencies from the English Lexicon Project (Balota et al., 2007) and investigated the semantic predictors in naming multisyllabic words. They found that words with denser semantic neighbourhoods and words with more meanings were recognised faster than those with sparser semantic neighbourhoods and fewer meanings. Furthermore, using Yap and Balota’s approach, Cortese and Schock (2013) found that the more imageable the disyllabic words, and the earlier they are acquired, the faster the naming latencies of disyllabic words. They concluded that semantic effects are larger in disyllabic words than monosyllabic words, as readers need more time to compute the pronunciation of the word, allowing semantics to affect the processing of words via interactive activation. In addition, spelling-to-sound correspondence is less predictable in disyllabic words than monosyllabic words, leading to more emphasis on semantics (cf. the AM hypothesis). It is therefore reasonable to assume that, if the semantic AoA effect is primarily evident in multisyllabic words, using a hybrid list of mono- and multisyllabic stimuli can lead to equivocal results.

Taken together, given that multisyllabic words are processed differently from monomorphemic words (e.g. Kuperman, Schreuder, Bertram & Baayen, 2009), results based on a heterogeneous set of items might not accurately reflect the contribution of AoA in word recognition. Additionally, the way in which compound words are recognized, either as whole words or via their individual morphemes, might have consequences for the type of AoA effects one can expect, with effects of the age at which the whole compound was learnt, the age at which the individual morphemes were learnt, and/or both.

One area of controversy with respect to the processing of compound words is whether the whole compound word or its individual morphemes drives the speed of access or recognition. Kuperman (2013) investigated the non-relational semantic properties for compound word recognition and whether the compound word or the individual meanings of the lexemes are accessed for compound word recognition. They used the rated imageability of the compound word and its morphemes to assess which of the measures would drive the lexical decision times obtained from the English Lexicon Project (Balota et al., 2007). The ratings of imageability were added to a baseline regression model that contained word length, individual lexeme frequency and compound word frequency. Kuperman found that compound word lexical decision times were predicted by compound word imageability but not morpheme imageability. In addition, Juhasz et al. (2015) extracted the reaction times of a subset of items (i.e. 629 compound words) from the English Lexicon Project and asked students to rate these based on imageability, AoA and familiarity of the compound word, together with the compound word’s semantic transparency and lexeme meaning dominance (LMD: the degree to which the meaning of a compound word is contained in its first and second lexeme). They found that the imageability, familiarity and AoA of the compound word predicted the reaction time of both the lexical decision and word naming tasks. In addition, morpheme frequency predicted the reaction time of both the lexical decision and word naming tasks. Semantic transparency predicted the speed of the lexical decision task but not the word naming task. However, there was no effect of AoA of the individual morphemes or the LMD on either task. Moreover, in a sentence reading task using eye-tracking, Juhasz (2018) assessed the role of AoA in compound words and found that the AoA of the compound word predicted fixation durations during sentence reading in gaze duration and total fixation duration, but the AoA of the individual morphemes showed no effect. Juhasz et al. (2015) concluded that the semantic representations of the morphemes are not automatically activated when the compound word is processed (cf. Kuperman, 2013). However, this does not necessarily indicate that lexical decomposition of compound words does not happen, as a large literature exist showing that the frequency of the morphemes can affect word naming and reading latencies (e.g. Juhasz, Starr, Inhoff & Placke, 2003; Juhasz et al., 2015; Juhasz, 2018).

Importantly, the studies detailed above require evaluation with regard to their generalisability. The semantic variables (e.g. imageability and familiarity) found by Juhasz and colleagues (Juhasz et al., 2015; Juhasz, 2018) play a greater role in lexical decision and text reading tasks than in word naming tasks, as the latter “has been repeatedly shown to be a more shallow task in that it does not implicate word semantics” (Kuperman, 2013, p.5). Put simply, a word naming task depends on a systematic mapping between phonology and orthography without access to semantics (Cortese et al., 2018; Kuperman, 2013; Snodgrass, 1984). This indicates that semantics may not be necessarily involved in spelling-sound correspondence.

## The present study

In the present study, we chose a word naming task, as it is a popular measure to assess AoA effects (e.g. Cortese et al., 2018; Gerhand & Barry, 1998; Ellis & Morrison, 1998; Morrison, Hirsh, Chappell & Ellis, 2002). Two experiments were conducted to evaluate the role of AoA in the processing of compound words and to investigate whether the AoA effect is found at the lexical/semantic level, as posited by Juhasz et al. (2015) and Juhasz (2018). If Juhasz and colleagues are correct, we would expect lexical/semantic variables such as familiarity and AoA to be significant determinants of the naming latencies. In addition, in line with Juhasz’s findings, we hypothesise that it is only the predictors of the compound word, not the predictors of individual morphemes that affect naming latencies. However, if word naming does not access semantics, as argued by Snodgrass (1984) and Kuperman (2013), there should be no effect from the semantic variables; only the AoA of the compound word should be found. This would partially support Juhasz et al. in that the AoA effect would be lexical but not necessarily or exclusively semantic.

Experiment 2 further investigated the lexical/semantic hypothesis of the AoA effect in compound words. We used a paradigm that had been used in lexical decision and eye-tracking studies (lexical decision: Libben et al., 2003; eye-tracking: e.g. Frisson, Niswander-Klement & Pollatsek, 2008) and presented the compound word with an additional space between the morphemes (e.g. *air plane*). Participants were asked to name the two words as a single word (i.e. to combine the two constituents). Presenting a compound as two separate words makes it more likely that each morpheme will be processed independently, at least initially, before being combined. The question of interest is whether readers would also extract the compound word meaning (cf. Brooks & Garcia, 2015), even though the task strictly speaking can be completed without this extra semantic processing step. If they do not combine the two morphemes and activate the compound, then we expect to only find AoA effects for the morphemes separately. However, if readers spontaneously combine the constituent words, then we would expect to (also) find AoA effects of the compound as a whole.

## Experiment 1: Word naming

This experiment was a partial replication of Juhasz et al.’s (2015) word naming experiment. However, in order to remove potential repetition effects, we excluded compound words that shared the same morpheme in the same position (e.g. *airplane* was used but words with air as the first morpheme such as *airport* were excluded). While Juhasz et al. (2015) did not include initial phoneme onset in their main analysis, they did include it in the supplementary analysis. We follow the supplementary analysis and included initial phoneme onset in the baseline model, as this is a powerful predictor of naming latencies.

### Method

#### Participants

To reduce experimenter bias, the data were analysed after all of the participants were recruited and a stopping rule was introduced for the two experiments. Based on Juhasz’s (2018) experiment, we used 48 British monolingual undergraduate students aged 18–20 years (M = 18.42 ± 0.64 years; seven males), who were given course credits (for reference, Juhasz, 2018, tested 45 American English students). The experiment was conducted in accordance with the British Psychological Society’s ethical guidelines and was approved by the University’s ethics committee. All participants had normal or corrected-to-normal vision and signed a consent form to participate in the study.

#### Materials

The stimuli consisted of 236 words which were primarily noun-noun compounds (see Supplementary Material). All words were taken from Juhasz et al.’s (2015) database of compound words. Repetition of the same morpheme within the same position was removed, as the repetition of items could lead to an increase in false positives (Winter, 2015). Each morpheme was therefore shown only once per position.

Word frequencies were extracted as Zipf values from the SUBTLEX-UK database (van Heuven, Mandera, Keuleers & Brysbaert, 2014) for compound word frequency and first and second morpheme frequency. According to Van Heuven et al. (2014), any words not found in SUBTLEX-UK were given a word frequency value of 0.696. This was given to only seven words (‘oxcart’, ‘prizefight’, ‘turtledove’, ‘castoff’, ‘carryall’, ‘filmstrip’ and ‘campground’).

From Juhasz et al.’s (2015) database, we used the ratings for compound word length, compound word AoA, semantic transparency (ST), compound word familiarity, compound word imageability and lexeme meaning dominance (LMD). Although Juhasz et al. (2015) tested the influence of the individual morphemes during a naming experiment, they did not include the ratings of those individual morphemes. We therefore obtained the mean familiarity for compound words and first and second morphemes from Balota, Pilotti and Cortese’s (2001) familiarity database using Davis’s (2005) N-Watch software. Using the AoA database from Cortese and colleagues (Cortese & Khanna, 2008; Schock et al. 2012b), we extracted the AoA ratings for the first and second morpheme. The AoA rating was log-transformed to make it an appropriate fit for the data, as AoA is not linearly related to lexical access, and to reduce the variance within AoA (Baayen, 2010). Referring to the imageability database from Cortese and colleagues (Cortese & Fugett, 2004; Schock et al. 2012a), we extracted the mean imageability for the first and second morpheme (See Table 1 for psycholinguistic characteristics).

**Table 1:** Descriptive statistics for word target characteristics for compound word

Predictors	Compound word			First morpheme			Second morpheme		
	M	SD	Range	M	SD	Range	M	SD	Range
Word length	8.61	1.34	6–13	4.42	1.02	2–8	4.19	0.84	2-7
Frequency (out of 7)	2.67	0.72	0.696–4.48	4.50	0.79	2.69-6.78	4.69	0.82	2.38-7.42^1^
Familiarity (out of 7)	5.77	1.15	1.57–7.00	4.72	1.66	1-6.41	4.98	1.50	1-6.43
Imageability (out of 7)	4.28	1.61	1.05–6.95	4.95	1.47	1.40-7.00	5.07	1.44	1.40-6.90
AoA (out of 7)	4.70	1.22	1.93–7.00	3.54	0.86	1.70-6.10	3.35	0.82	2-6.30
ST (out of 7)	4.59	1.33	1.6–6.71	NA				NA	
LMD (out of 10)	5.17	1.42	1.47–8.67	NA				NA	

*AoA* Age-of-Acquisition, *ST* Semantic Transparency, *LMD* Lexeme Meaning Dominance

^1^ Although there is a discrepancy between the maximum and range of values shown in this table, this discrepancy is from the van Heuven et al.'s SUBTLEX-UK online database and the Likert scale used (scale of 1-7 discussed on their website). We had used the word "to", which had a Zipf scale of 7.42 and function words tend to go beyond the maximum score

Each word in the data set was coded dichotomously (1 or 0) according to the following 11 categories, where 1 states the presence of the feature and 0 defines the absence of a feature: bilabial, labiodental, dental, labiovelar, postalveolar, alveolar, palatal, palatal alveolar, glottal, velar and voiced. These features are known to be very powerful in predicting naming response latencies (Spieler & Balota, 1997; Treiman et al., 1995).

#### Procedure

Participants were tested individually in front of a computer screen with a microphone approximately 15 cm away from the mouth. They were instructed to name the word as fast as possible without compromising their accuracy and E-prime software (E-studio, E-Prime 2.0) was used to collect the responses. A fixation cross appeared at the centre of the screen for 250 ms, after which the stimulus appeared in the same position as the fixation cross, which was shown until the word was named or 2,000 ms had passed. Stimuli were presented in uppercase using Arial font (size: 34). This was followed by an inter-trial interval of 1,000 ms. Each session lasted approximately 10 minutes.

### Results

A GLMM was conducted on the reaction time data using the lme4 package (Bates, Maechler & Dai, 2010) within R statistical programming open code software (R Development Core Team, 2017). It was not conducted on accuracy, as the results were at ceiling and the mean proportion correct was 0.987 (SD = 0.11). Error rates, missed/late responses, reaction times less than 200 ms and reaction times 2.5 SD above or below the participant mean were removed from the analysis. This led to a total of 6.90% responses being removed. The GLMM included random intercepts and slopes to reduce type I and II error rates (Barr, Levy, Scheepers & Tily, 2013; Schielzeth & Forstmeier, 2008). Finally, the Variance Inflation Factor (VIF) was calculated to approximate the influence of multi-collinearity on the regression coefficients. A VIF larger than 5 suggests moderate influence, larger than 10 is seen as a strong indicator of multicollinearity (Fox & Weisberg, 2010).

In order to check for AoA effects and the extent to which the AoA of the compound word and its morphemes could explain the variance beyond the main linguistic processing predictors (without the fear of collinearity), we used a benchmark model advocated by Kuperman (2013), Juhasz et al. (2015) and Juhasz (2018). This benchmark GLMM included whole compound word frequency, compound word length, morpheme 1 frequency and morpheme 2 frequency. We also included phoneme onset in the baseline model (i.e. bilabial, labiodental, dental, labiovelar, postalveolar, alveolar, palatal, palatal alveolar, glottal, velar and voiced) to examine whether the effects of AoA shown were genuine and not a result of initial phoneme onset. Each variable of interest was added separately to the model for the log-transformed reaction time. All predictor variables, excluding those for initial phoneme onset, were centred on their means. Following Nakagawa and Schielzeth (2013), marginal and conditional R^2^ values were obtained with the r.squared GLMM() function of the MuMIn package (Barton & Barton, 2015). The marginal R^2^ is an estimate of the variance explained by fixed factors only, while the conditional R^2^ explains the variance of the whole model (i.e. fixed and random factors). The maximum VIF for each of these models was 6.15, which was driven by the alveolar initial phoneme onset. The alveolar initial phoneme onset was therefore removed from the analyses.^1^ This led to a VIF factor for each model of around 1.55. T values were computed for each variable of interest. Variables were significant at the alpha = .05 level if the absolute *t* value was greater than 2.00 (Baayen, Davidson & Bates, 2008). The correlation matrix between the variables is shown in Table 2.

**Table 2:** Correlations between independent variables

	CL	CF	CFA	CAOA	CI	ST	LMD	FML	FMF	FMFA	FMAoA	FMI	SML	SMF	SMFA	SMAOA
CF	0.01															
CFA	0.07	0.33^***^														
CAoA	0.04	-0.20^**^	-0.65^***^													
CI	0.04	0.04	0.54^***^	-0.66^***^												
ST	0.08	-0.08	0.37^***^	-0.37^***^	0.45^***^											
LMD	-0.06	-0.01	0.00	-0.06	0.24^***^	0.03										
FML	0.78^***^	-0.02	0.05	-0.02	0.04	0.04	-0.07									
FMF	-0.08	0.27^***^	0.22^***^	-0.11	-0.10	0.11	-0.10	-0.11								
FMFA	0.06	0.09	0.19^**^	-0.17^**^	0.13^+^	0.21^**^	-0.09	0.08	0.49^***^							
FMAoA	0.23^***^	-0.09	-0.15^*^	0.30^***^	-0.12^+^	-0.16^*^	0.00	0.28^***^	-0.59^***^	-0.50^***^						
FMI	0.10	-0.13^+^	0.03	-0.24^***^	0.42^***^	0.22^***^	0.08	0.14^*^	-0.13^+^	0.18^**^	-0.31^***^					
SML	0.65^***^	0.04	0.14^*^	0.09	0.01	0.08	0.01	0.03	0.00	0.00	0.03	-0.01				
SMF	-0.12^+^	0.15^*^	0.00	-0.01	-0.15^*^	0.09	-0.17^**^	0.02	0.21^***^	0.13^+^	-0.06	-0.19^**^	-0.21^***^			
SMFA	0.01	0.09	0.10	-0.02	-0.01	0.23^***^	0.06	0.03	0.05	0.09	-0.02	-0.01	-0.02	0.55^***^		
SMAoA	0.17^*^	0.01	-0.11	0.08	-0.12^+^	-0.08	-0.14^*^	0.02	-0.05	-0.05	0.11	-0.07	0.24^***^	-0.55^***^	-0.41^***^	
SMI	0.03	-0.09	0.14^*^	-0.20^***^	0.48^***^	0.15^*^	0.38^***^	-0.07	-0.19^**^	-0.05	-0.07	0.34^***^	0.14^*^	-0.25^***^	0.13^*^	-0.34^***^

*CL* compound word length, *CF* compound word frequency, *CFA* compound word familiarity, *CAoA* compound age-of-acquisition, *CI* compound word imageability, *ST* semantic transparency, *LMD* lexeme meaning dominance, *FML* first morpheme length, *FMF* first morpheme frequency, *FMAoA* first morpheme age-of-acquisition, *FMFA* first morpheme familiarity, *FMI* first morpheme imageability, *SML* second morpheme length, *SMF* second morpheme frequency, *SMFA* second morpheme familiarity, *SMAoA* second morpheme age-of-acquisition, *SMI* second morpheme imageability

^+^p < .10, * p < .05, ** p < .01 and *** p < .001

The mean reaction time for word naming was 495 ms (SD =116; 95% CI [461–528]). The compound word AoA measure affected the naming latency when added to the baseline, including compound word length, frequency, morpheme frequencies and initial phoneme onset (*b* = 0.14, *t* = 2.42, *p* = .016). Early acquired compound words were named faster than late acquired compound words. The effect of LMD approached significance (*b* = -0.01, *t* = -1.91, *p* = .057). The naming of the compound word was faster and driven by the individual left morpheme of the compound word in contrast to the individual right morpheme of the compound word. There were no significant effects on naming latencies for compound word length, compound word frequency, the frequency of the first- and second morpheme, compound word familiarity, compound word imageability, semantic transparency, familiarity, and AoA and imageability of the first and second morpheme. Tables 3 and 4 show the coefficients, standard errors and *t* values for each variable of interest for the baseline model and other models when added to the baseline model.

**Table 3:** Linear mixed-effects regression results for the baseline model for Experiment 1^a^

Values	CL	CFreq	FFreq	SFreq	Bilabial	Labiodental	Dental	Labiovelar	Postalveolar	Palatal	PA	Glottal	Velar	Voiced
Coefficient	0.005	-0.015	0.003	-0.003	0.082	0.026	-0.010	0.014	-0.020	-0.030	-0.030	0.020	0.049	0.035
SE	0.006	0.010	0.010	0.009	0.011	0.015	0.039	0.012	0.025	0.055	0.021	0.014	0.012	0.009
95% CI -	-0.005	-0.036	-0.017	-0.021	0.060	-0.004	-0.087	-0.092	-0.070	-0.136	-0.072	-0.007	0.025	0.017
95% CI +	0.017	0.005	0.021	0.015	0.104	0.056	0.067	0.037	0.029	0.081	0.012	0.047	0.072	0.042
t-value	0.99	-1.45	0.27	-0.29	7.37*	1.73	-0.25	1.19	-0.78	-0.50	-1.42	1.43	4.09*	3.90*
R^2^(m) R^2^(c)	0.111 0.484													

**Note:** Coefficients and standard error are presented for each variable in the baseline model: compound word length (CL), compound word frequency (CFreq), morpheme 1 frequency (FFreq), morpheme 2 frequency (SFreq) and bilabial, labiodental, dental, labioveolar, veolar, postalalveolar, palatal, palatal.alveolar (PA), glottal, velar and voiced, with the alveolar factor being removed as a result of its VIF being above 3, indicating moderate co-linearity

95% CI - = lower confidence interval; 95% CI+ = upper confidence Interval and SE = standard error

*Significant at the α = .05 level

^a^This model did not converge with the variable of interest. We only included a random subject and item intercept

**Table 4:** Linear mixed effects regression results for the 11 variables of interest for Experiment 1

Values	CFAa	CAoAa	CIa	STa	LMDa	FMFAa	FMAoAa	FMIb	SMFAb	SMAOAa	SMIa
Coefficient	-0.010	0.141	-0.005	-0.002	-0.010	0.002	-0.022	0.001	-0.007	0.018	-0.008
SE	0.007	0.058	0.005	0.006	0.005	0.005	0.091	0.005	0.006	0.089	0.005
95% CI -	-0.024	0.025	-0.015	-0.014	-0.02	-0.012	-0.201	-0.009	-0.018	-0.160	-0.018
95% CI +	0.004	0.257	0.005	0.010	0.00	0.008	0.156	0.011	0.005	0.196	0.002
t-value	-1.38	2.43*	-1.02	-0.33	-1.91	0.36	-0.25	0.23	-1.23	0.21	-1.48
R^2^(m)	0.112	0.116	0.111	0.111	0.114	0.112	0.111	0.111	0.113	0.111	0.113
R^2^(c)	0.484	0.484	0.483	0.484	0.484	0.484	0.485	0.484	0.486	0.485	0.484

**Note:** Coefficients and standard error are presented for each variable when added separately to the baseline model containing morpheme 2 length, frequency, morpheme 1 frequency, morpheme 2 frequency and initial phoneme onset, which includes bilabial, labiodental, dental, labioveolar, veolar, postalalveolar, palatal, palatal.alveolar, glottal, velar and voiced, with the alveolar factor being removed as a result of its VIF being above 3, indicating moderate co-linearity

95% CI - = Lower confidence interval; 95% CI+ = Upper confidence Interval

*SE* standard error; *CFA* compound word familiarity, *CAoA* compound word age of acquisition, *CI* compound word imageability, *ST* semantic transparency, *LMD* lexeme meaning dominance; *FMFA* first morpheme familiarity, *FMAoA* first morpheme age of acquisition, *SMI* first morpheme imageability, *SMFA* second morpheme familiarity, *SMAoA* second morpheme age of acquisition, *SMI* second morpheme imageability

*Significant at the α = .05 level

^a^This model did not converge. For these analyses, a by-subject random slope was only included for the variable of interest

^b^This model did not converge with the variable of interest. We only included a random subject and item intercept

### Discussion

Experiment 1 found that the AoA of the compound word affected word naming with no effects from the AoA of individual morphemes. Early acquired words were named faster than late-acquired words. This is in line with the literature on the effects of AoA on monomorphemic word naming (e.g. Ellis & Morrison, 1998; Preece, 2015), bisyllabic word naming (Cortese & Schock, 2013) and compound word naming (e.g. Juhasz et al., 2015). This indicates that the compound word was processed as a whole word.

However, it is surprising that semantic measures such as imageability and familiarity did not predict compound word naming latencies, since previous research (Cortese & Schock, 2013; Juhasz et al., 2015; Yap & Balota, 2009) has shown that semantic measures (e.g. number of senses, semantic neighbourhood size, imageability and familiarity) predicted the speed of naming multi-syllabic words. Cortese and Schock (2013) argued that semantic activation can affect the generation of a phonological code and that semantics plays a larger role in polysyllabic word recognition than in monosyllabic word recognition. However, the discrepancy between the current study and prior research is unclear.^2^

We did not find any effects of morpheme frequency on compound word naming latencies. This contradicts prior research such as that of Juhasz et al. (2003), who showed that participants responded faster to the compound word when the ending lexeme was high- as opposed to low-frequency. Moreover, the beginning morpheme effect was shown only for the word naming task, but not in other tasks such as a lexical decision task or sentence reading. However, our findings showed a reduced word frequency effect in naming. This may indicate that during word naming experiments in which participants need to read words quickly, semantic involvement is less pertinent compared to natural reading or a situation in which a decision needs to be made whether the letter string is an existing word or not (LDT). If this is the case, and assuming that frequency effects are at least partially semantically driven, then the absence of a compound word and/or lexeme frequency effect might be task-related. The implications of these findings will be discussed in the General Discussion in relation to AoA effects.

## Experiment 2: Combinatorial naming

The aim of this experiment was to test whether the effects of AoA are present when compound words are presented with a space between the two morphemes. This method of presentation arguably induces initial decomposed processing of the compound and we therefore expected an effect of the frequency of the morphemes to become apparent. In addition, if combinatorial naming reflects morphological decomposition, we would also expect AoA effects for the individual morphemes to be demonstrated. Furthermore, when the compound word has a space between the two words, the two morphemes would need to combine for the effects of compound word meaning to occur, as the meaning of the morphemes would be compared to the meaning of the compound word (cf. Brooks & de Garcia, 2015; Kuperman, 2013). This extra semantic step that participants are likely to make in this paradigm (at least if they extract the compound’s meaning as well) might result in semantic variables such as imageability and familiarity to become more influential, especially for the first lexeme (Juhasz et al., 2003), as participants might start naming the first lexeme before the second has been fully processed. Circumstantial evidence that semantics might be more involved when a compound is broken up comes from Frisson et al. (2008). They found that semantic transparency of English compounds did not affect any of the eye-movement measures when the compounds were presented unspaced (see also Pollatsek & Hyönä, 2005, for evidence from Finnish). However, when the same compounds were presented with a space, transparency effects did emerge, with opaque compounds taking longer to process than transparent ones. While Frisson et al. did not examine other semantic variables except for transparency, their results do suggest that a minimal change in presentation can modify the way compounds are processed semantically.

### Method

#### Participants

Forty-eight British monolingual undergraduate students aged 18–20 years (M =18.38 ± 0.60 years; four males) participated in the study and were remunerated with course credits. The experiment was conducted in accordance with British Psychological Society ethical guidelines and was approved by the University’s ethics committee. All participants had normal or corrected-to-normal vision and signed a consent form to participate in the study. None of the students participated in the previous experiment.

#### Materials and procedures

The same materials and procedures were used as in Experiment 1 with the following exception: a space was inserted between the two morphemes of the compound (e.g. air plane). Participants were informed that they would be presented with two lexical strings which they had to name as one word.

### Results

We used the same analysis as in Experiment 1. The analysis was not conducted on accuracy, as the results were at ceiling and the mean proportion correct was 0.995 (SD = 0.07). Error rates, missed/late responses, reaction times less than 200 ms and reaction times that were 2.5 SD above or below the participant mean were removed from the analysis, leading to 5.04% of the responses being removed. The maximum VIF for each of the models was 6.15, which was driven by the alveolar initial phoneme onset. The alveolar initial phoneme onset was removed from the analyses^3^. This led to a VIF factor for each model of 1.56. The mean reaction time for combinatorial naming was 495 ms (SD =104; 95% CI [465–526]), which was similar to the naming latencies for the word naming task in Experiment 1 (*t* < 1). The compound word frequency predicted combinatorial naming (*b* = -0.008, *t* = -2.14, p = 0.03), while the effects of compound word length approached significance (*b* = 0.004, *t* = 1.85, *p* = .07). The longer the compound word, the slower the reaction times to combine the morphemes to form a compound word. The compound word familiarity was a strong predictor of combinatorial naming (*b* = -0.01, *t* = -4.53, *p* < .001) when added to the baseline, which included compound word length, frequency, morpheme frequencies and initial phoneme onset. The more familiar the compound word, the faster the combinatorial naming latencies.

In addition, the compound word AoA was a good predictor of combinatorial naming (*b* = 0.10, *t* = 4.71, *p* < .001), as was compound word imageability (*b* = -0.07, *t* = -3.95, *p* < .001), the LMD (*b* = -0.006 , *t* = -2.94, *p* = .004), the AoA of the first and second morpheme (*b* = 0.08, *t* = 2.09, *p* = .04 and *b* = 0.09, *t* = 2.80, *p* = .006, respectively) and the imageability of the first and second morpheme (*b* = -0.006, *t* = -3.15 , *p* = .002, and *b* = -0.006, *t* = -3.04, *p* = .003, respectively). Put simply, the earlier the compound word and the individual morphemes were acquired, and the more imageable the compound word and its individual morphemes, the faster the combinatorial naming latencies. Furthermore, the more the right morpheme contributes to the meaning of the compound word, the faster the combinatorial naming latencies. There were no significant effects on naming latencies for compound word length, the frequency of the first and second morpheme, semantic transparency, or the familiarity of the first and second morpheme. Tables 5 and 6 show the coefficients, standard errors and *t* values for each variable of interest for the baseline model and other models when added to the baseline model.

**Table 5:** Linear mixed effects regression results for the baseline model for Experiment 2a

Values	CL	CFreq	FFreq	SFreq	Bilabial	Labiodental	Dental	Labiovelar	postalveolar	Palatal	PA	Glottal	Velar	Voiced
Coefficient	0.004	-0.008	-0.005	-0.005	0.019	0.031	0.016	0.017	-0.017	0.004	-0.006	0.018	0.003	0.011
SE	0.002	0.004	0.003	0.003	0.004	0.006	0.015	0.004	0.009	0.020	0.008	0.005	0.004	0.003
95% CI -	-0.000	-0.016	-0.013	-0.001	0.011	0.023	-0.009	-0.013	-0.031	-0.038	-0.021	0.011	0.004	0.006
95% CI +	0.008	0.000	0.001	0.017	0.026	0.044	0.046	0.032	0.004	0.039	0.009	0.031	0.012	0.020
t-value	1.85	-2.14*	-1.37	-1.54	4.55*	5.54*	1.07	3.97*	-1.86	0.18	-0.75	3.57*	0.67	3.19*
R^2^(m)	0.015 0.408													
R^2^(c)														

**Note:** Coefficients and standard error are presented for each variable in the baseline model: compound word length (CL), compound word frequency (CFreq), morpheme 1 frequency (FFreq), morpheme 2 frequency (SFreq) and bilabial, labiodental, dental, labioveolar, veolar, postalalveolar, palatal, palatal.alveolar (PA), glottal, velar and voiced, with the alveolar factor being removed as a result of its VIF being above 3, indicating moderate co-linearity. 95% CI - = Lower confidence interval; 95% CI+ = Upper confidence Interval and SE = standard error

*Significant at the α = .05 level

^a^This model did not converge with the variable of interest. We only included a random subject- and item intercept

**Table 6:** Linear mixed effects regression results for the 11 variables of interest for Experiment 2

Values	CFAb	CAoAa	CIa	STa	LMDb	FMFAb	FMAoAa	FMIa	SMFAb	SMAoAa	SMIb
Coefficient	-0.010	0.098	-0.006	-0.003	-0.006	-0.001	0.077	-0.006	-0.003	0.092	-0.006
SE	0.002	0.021	0.002	0.002	0.002	0.002	0.037	0.002	0.002	0.033	0.002
95% CI -	-0.012	0.056	-0.009	-0.007	-0.010	-0.005	0.003	-0.010	-0.007	0.026	-0.009
95% CI +	-0.006	0.014	-0.003	0.001	-0.002	0.003	0.151	-0.003	0.001	0.158	-0.002
t-value	-4.53*	4.71*	-3.95*	-1.49	-2.94*	-0.29	2.09*	-3.15*	-1.28	2.80*	-3.04*
R^2^(m)	0.018	0.018	0.017	0.015	0.016	0.015	0.015	0.016	0.015	0.016	0.016
R^2^(c)	0.408	0.408	0.408	0.408	0.408	0.408	0.411	0.408	0.408	0.408	0.408

95% CI - = Lower confidence interval; 95% CI+ = Upper confidence Interval

*SE* standard error; *CFA* compound word familiarity, *CAoA* compound word age of acquisition, *CI* compound word imageability, *ST* semantic transparency, *LMD* lexeme meaning dominance; *FMFA* first morpheme familiarity, *FMAoA* first morpheme age of acquisition, *FMI* first morpheme imageability, *SMFA* second morpheme familiarity, *SMAoA* second morpheme age of acquisition, *SMI* second morpheme imageability

*Significant at the α .05 level

^a^This model did not converge. For these analyses, a by-subject random slope was only included for the variable of interest

^b^This model did not converge with the variable of interest. We only included a random subject- and item intercept

### Discussion

In the second experiment, we found a significant effect of the compound word and morpheme AoA, together with several other psycholinguistic variables (e.g. familiarity, imageability, LMD and the imageability of the first and second morpheme). The pattern shown in Experiment 2 partially replicates the pattern that Juhasz et al.’s (2015) reported. However, the current study differs from previous studies, as effects of lexeme frequency were not shown and, unlike Juhasz et al.’s study, we showed effects of morpheme AoA and imageability, together with LMD. These results indicate that the AoA of the compound as a whole affects processing, even when compound recognition is forced down the morphemic decomposition route. This suggests that, even when the task did not require participants to (re-)combine the constituent words, they nevertheless spontaneously did so. In addition, and in contrast to Experiment 1, we also found AoA effects for the individual morphemes, indicating that when the constituent words are presented with a space, they are processed separately as well to a degree.

The finding that both whole compound and first and second morpheme imageability affected processing indicates that both were processed to a semantic level (contra Kuperman, 2013). We suggest that when the compound is presented with a space, participants focused more on the morphemes than in Experiment 1, leading to certain semantic properties of the morphemes to be activated automatically. However, the results indicate that in addition to processing the morphemes in isolation, participants also combined them and accessed the semantic representation of the whole compound.

Although the present study observed an effect of compound word familiarity, we found no effects of the familiarity of the individual morphemes in the combinatorial naming task. This discrepancy could result from the corpora used. For the compound word, we used Juhasz et al.’s familiarity measure, while for the individual morphemes, we used the familiarity scores from Balota et al. (2001).^4^ Juhasz et al. asked participants to rate the familiarity of the compound word based on its meaning and frequency, whereas Balota et al. required participants to rate words solely on their frequency. This suggests that Juhasz et al.’s familiarity measure may not only be affected by subjective frequency but also semantics, whereas Balota et al.’s familiarity measure is primarily influenced by subjective frequency. This interpretation is supported by the current study. As shown in Table 2, the familiarity of the individual morphemes from Balota et al. weakly correlated with their imageability (*r* = .18 and .13 for the first and second morpheme respectively; cf. *r* = .28 between subjective frequency and meaningfulness in Balota et al.), while the familiarity of the compound word strongly linked to compound word imageability (*r* = .54; cf. *r* = .48 between compound word imageability and familiarity in Juhasz et al.). Hence, the compound word familiarity effect again supports the conclusion that the compound was (also) processed as a whole and that semantics was involved in this process. In contrast, the absence of a familiarity effect for the constituents is more in line with the absence of a morpheme frequency effect, likely due to the two variables – morpheme frequency and Balota et al.’s familiarity ratings – tapping into similar concepts (see also Table 2).

It is noteworthy that compound word frequency affected the naming latencies in Experiment 2, but not in Experiment 1, and that the beginning lexeme frequency was not a significant predictor of naming latencies. One explanation could be that the lexical-semantic representation is accessed without the frequency of the individual morphemes influencing the naming latencies. Given Inhoff, Starr, Solomon and Placke’s (2008) finding that the beginning morpheme frequency effect may be modulated by the semantic overlap between the beginning lexeme and the compound word, it is likely that these frequency effects reside at the semantic level in compound words at least.

## General discussion

This study is the first to assess the effects of AoA on compound words through word naming and combinatorial naming. In both experiments, the results showed that the age at which a person learnt a compound word significantly impacted on the naming latencies. Our first study partially supports the findings of Juhasz et al. (2015), as the whole-word AoA was a predictor of word naming. However, we did not find the effects of imageability and familiarity shown in Juhasz et al. This indicates that within this task, the AoA effect is lexical in nature, rather than semantic. Within the second experiment only, semantic predictors (i.e. imageability and familiarity) were also found to have a significant impact on naming latencies. This difference in the findings between Experiments 1 and 2 indicates that the results of the first experiment were not due to item selection or the use of a different baseline model. We posit that the differences in effects of semantic variables are due to the different processing that is required in the second task where participants are encouraged to combine the two morphemes in a manner that demands the utilisation of extra semantic resources (e.g. Brooks & de Garcia, 2015; Kuperman, 2013).

The semantic locus theory predicts that AoA effects would be shown in tasks that focus primarily on semantic processing. Although the results of the current combinatorial naming experiment (Experiment 2) support the semantic hypothesis for the AoA effects in compound words, the results of the compound word naming experiment (Experiment 1) contradict this hypothesis. The former focuses on orthographic-phonological mapping with access to semantics as a result of the space being interjected between morphemes, while the latter focuses on orthographic-phonological mapping without access to semantics (Cortese et al., 2018; Kuperman, 2013; Snodgrass, 1984). This does not mean that we want to argue against a semantic level of the AoA effects during the natural processing of compound words, but that a semantic locus may not be necessary to produce an AoA effect, reflecting the argument for monomorphemic word processing proposed by Monaghan and Ellis (2010) and Joseph et al. (2014). If anything, the AoA effects are found at least at the lexical level, supporting the argument by Juhasz et al. (2015) that the AoA effects reflect the lexical aspects of compound word recognition since they gauge a reader’s past experience with both a word’s form and its meaning. In addition, the findings of Juhasz (2018) can be subsumed under a multiple-loci account. Furthermore, the present and prior results can extend the multiple-loci account of the AoA effects found for monomorphemic words (Catling & Johnston, 2009; Moore et al., 2004) to complex words such as compound words.

The Arbitrary Mapping (AM) hypothesis (e.g. Ellis & Lambon Ralph, 2000) is based on the concept that early-acquired items entering the training network and benefiting from its plasticity. Early-acquired items begin to adjust the connections or weights for the network. This makes the network lose plasticity, giving early-acquired items a processing advantage over late-acquired items, making the latter difficult to consolidate. The AM hypothesis asserts that, AoA effects would be present but weaker in a word naming task than in a picture naming task as the mapping between the input (orthography) and output (phonology) is more straightforward (cf. Lambon Ralph & Ehsan, 2006) and can be done without reference to semantics. However, the AM hypothesis would not make predictions about any differences in AoA effects across the two experiments – simply that we should expect them to be apparent in both (which was the case in the current study). This raises interesting further research questions as to whether the AoA effect would generalise to a transparent (non-arbitrary) language (e.g. Spanish) – whether the individual morphemes (*quitar* and *sol* in Spanish) would contribute to the meaning of the compound word (e.g. *quitasol*) or whether the compound word would be processed solely at a lexical level. Finally, the AM hypothesis was derived from computational modelling studies (Ellis & Lambon Ralph, 2000; Lambon Ralph & Ehsan, 2006; Monaghan & Ellis, 2010), allowing researchers to make and experimentally test finer predictions about the effects of AoA. However, the computational modelling of the AM hypothesis was limited to monomorphemic words and has not been expanded to include complex words, specifically compound words. An AM hypothesis derived from computational modelling studies in complex words would enable researchers to see how the effects of AoA are processed, structured and, more interestingly, how they may be learnt.

We found that the naming latencies for the combinatorial naming task were similar to the naming latencies for the standard word naming task. This indicates that combinatorial naming was not more difficult than the standard word naming task, although the predictors for both were different. One explanation for this could be that word naming depends on a translation between orthography and phonology without access to semantics (Kuperman, 2013; Snodgrass, 1984). Kuperman (2013) argues that a simple word embedded in a compound word as a morpheme forces the simple word to lose its semantic influence, thus the morphemes are not semantically accessed during compound recognition (see also Frisson et al., 2008). The main process of reading is to arrive at the whole-word/semantic level to allow for efficient processing to enable higher-level cognitive mechanisms (e.g. reading comprehension) to take place (Nation, 2017). For instance, *firefly* is a type of insect. However, dividing the compound word into morphemes with a space (*fire fly*) would lead to inefficient processing and ambiguity, as it could suggest shooting the fly, which is far from the whole-word meaning of *firefly*, the correct interpretation. It is therefore important for morphemes not compete with the whole word to reduce ambiguity.

This similarity in the naming latencies between the two experiments also contradicts research from Juhasz, Inhoff and Rayner (2005), who used a lexical decision task and found that inserting a space between compound words led to slower reaction times than with no space, as it disrupted the processing. However, Inhoff, Radach and Heller (2000) found that inserting a space in the compound word led to faster naming latencies. Task-related discrepancies may explain the discrepant findings of Juhasz et al. (2005) and the current study. The lexical decision task involves a decision process, not otherwise included in natural reading. In addition, Schilling, Rayner and Chumbley (1998) found that there was a positive correlation between total reading times and a lexical decision task, as opposed to a naming task that had a positive relationship with first fixation and gaze duration. This indicates that the lexical decision task assesses the later processes of reading, while naming measures the earlier processes and that inserting a space may have no influence on the earlier processes of word reading but may affect the later processes of reading. The current study also included words with and without a space in separate experiments; they were not placed in the same list, as opposed to Inhoff et al. This could have led to different cognitive strategies for the participants or to participants being confused why some compounds were spelled with a space and others not. Future research should investigate the production of compound words (i.e. with and without a space) as related to list composition.

One other finding of interest was that Lexeme Meaning Dominance (LMD) was a significant predictor of naming latencies in Experiment 2. This was surprising, as Juhasz et al. (2015) did not find that LMD affected the latencies in their naming and lexical decision tasks. We would argue that the space interjected between the morphemes captures the reader’s attention, facilitating an increase in morphemic activation. This would encourage participants to access the semantic properties of the morphemes, which in turn can be compared to the meaning of the compound word as a whole. This comparison might involve assessing whether there are overlapping similarity relations (e.g. how similar is *sun* to *sunshine*?), conceptual relations (how are *sun* and *shine* related to *sunshine*?) or another kind of semantic association. The present study asked participants to merge the two morphemes to form a compound word (e.g. to read *sun* and *shine* as one word and to verbalise it as *sunshine*), which could have led to stronger semantic effects. This can explain why LMD and the imageability and AoA of the morphemes affected naming latencies when LMD was not found to affect whole compound word recognition and naming (Inhoff et al., 2008; Juhasz et al., 2015; Kuperman, 2013).

However, this does not explain why semantic transparency was not shown. While this is in line with Frisson et al. (2008) reading study when compounds were presented unspaced, the authors did observe a (slightly delayed) transparency effect when the same compounds appeared with a space. In general, their results, together with the present results, suggest that semantic effects are more prevalent when compounds are presented with a space rather than unspaced. However, the contribution of the different semantic variables seems to differ. One explanation, in addition to differences in the tasks used, could be that semantic transparency is a corrective measure to reduce any accidental activation from the meaning of the morphemes that could incorrectly contribute to the meaning of the compound word (Kuperman, 2013). Our findings attempt to explain why individual morpheme predictors, such as morpheme AoA and imageability, were found to affect naming latencies in the present study, but perhaps not in other studies such as Juhasz et al. (2015). Although these standard word naming and combinatorial naming tasks differ only by one trivial detail at face value, they force participants to activate lexical predictors either with or without semantic predictors. It is important to “note that as the sample [size] of each experiment was moderate and the designs were also slightly different” (Elsherif, Sahan & Rotshtein, 2017, p.26), we should still remain cautious about these effects. It would therefore be beneficial not only to replicate these results, but also to use them for further investigation into the loci and processing of the AoA effects, as well as using other measures (e.g. duration of participants’ utterances) to corroborate these findings.^5^

Overall, we found that the AoA of the compound word drove the naming latencies of compound words in both experiments (i.e. whole word and combinatorial processing), together with the AoA effects of the individual morphemes in combinatorial naming. This indicates that the AoA effects are lexical in nature. This partly supports Juhasz and colleagues’ (Juhasz et al., 2015; Juhasz, 2018) view that the AoA effects for complex words is lexical, but not semantic, in nature. Furthermore, these results extend the findings of monomorphemic word processing to compound words. To sum up, the effect of compound word AoA is more pervasive than originally thought, as it exists not only in monomorphemic but also morphologically complex words. In addition, morphemic AoA, along with the whole word AoA and semantic effects becomes apparent when undertaking a task that combines separate morphemes into a single compound word.

## Electronic supplementary material

ESM 1 (XLSX 49.9 kb)

## Footnotes

1 The patterns of the results did not change when alveolar was included or excluded as a fixed predictor.

2 To check whether the findings were due to regional differences, we gave 20 university of Birmingham students a ratings scale to measure familiarity and imageability of the compound word. We used the same instructions as Juhasz et al. (2015). Participants rated if they knew the meaning of the word and used it frequently. The scale was from 1 to 7, where higher numbers indicate higher familiarity for an item. For the imageability rating, we paraphrased the instructions from those of Schock, Cortese, and Khanna (2012) as per Juhasz et al.’s instructions: (1) the number of items was removed, (2) examples used in Schock et al. were changed to the words *blackboard* and *something*, (3) instructions were added to illustrate high- and low-imagery compound items (see supplementary material for questionnaires). The scale for the imageability rating ranged from 1 to 7, where higher numbers indicate greater ease of imagery for an item. We found our ratings to be similar to the American ratings. We also used our ratings into our model and found no significant effect of familiarity or imageability on naming latencies, indicating that the results were not due to regional differences. In our analyses, we retained Juhasz et al.’s familiarity and imageability ratings for compound words.

3 The patterns of the results did not change when alveolar was included or excluded as a fixed predictor.

4 We did not use Balota et al.’s (2001) familiarity scores for the compound words, since they are specifically limited to monomorphemic words.

5 We thank an anonymous reviewer for this suggestion.
